# Electrochemical Synthesis of Novel Zn-Doped TiO_2_ Nanotube/ZnO Nanoflake Heterostructure with Enhanced DSSC Efficiency

**DOI:** 10.1007/s40820-016-0099-z

**Published:** 2016-07-02

**Authors:** Aijo John K, Johns Naduvath, Sudhanshu Mallick, Jacob W. Pledger, S. K. Remillard, P. A. DeYoung, Manju Thankamoniamma, T. Shripathi, Rachel Reena Philip

**Affiliations:** 1 Department of Physics Union Christian College, Aluva, Kerala India; 2 Department of Metallurgical Engineering and Material Science, Indian Institute of Technology, Mumbai, Maharashtra India; 3 Department of Physics, Hope College, Holland, MI 49423 USA; 4 Department of Physics, Sree Sankara College, Kalady, Kerala India; 5 UGC-DAE Consortium for Scientific Research, Indore, Madhya Pradesh India; 6 Present Address: Department of Physics, St. Thomas College, Thrissur, Kerala India

**Keywords:** Zn-doped TiO_2_ nanotubes, ZnO nanoflakes, Heterostructures, DSSC

## Abstract

**Abstract:**

The paper reports the fabrication of Zn-doped TiO_2_ nanotubes (Zn-TONT)/ZnO nanoflakes heterostructure for the first time, which shows improved performance as a photoanode in dye-sensitized solar cell (DSSC). The layered structure of this novel nanoporous structure has been analyzed unambiguously by Rutherford backscattering spectroscopy, scanning electron microscopy, and X-ray diffractometer. The cell using the heterostructure as photoanode manifests an enhancement of about an order in the magnitude of the short circuit current and a seven-fold increase in efficiency, over pure TiO_2_ photoanodes. Characterizations further reveal that the Zn-TONT is preferentially oriented in [001] direction and there is a Ti metal-depleted interface layer which leads to better band alignment in DSSC.

**Graphical Abstract:**

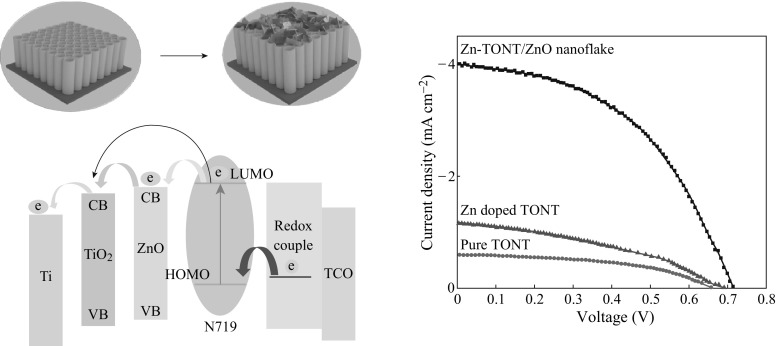

## Introduction

The ability to fine tune the optoelectronic and transport properties of the metal oxide semiconductors nominates them as promising candidates for chemical gas sensing, photocatalysis, energy conversion, and storage applications [1]. Among the metal oxide semiconductors, TiO_2_ and ZnO have been studied extensively in the recent years owing to their exceptional characteristics such as high stability, low-cost fabrication, nontoxicity, and excellent photoelectrochemical properties [2–5]. Nevertheless, these pure metal oxides individually exhibit relatively low energy conversion efficiencies because of the limited photoresponse range and the fast recombination rate of the generated charge carriers [6–9].

These drawbacks of the pure metal oxide semiconductors have led researchers to explore the heterostructures using TiO_2_ and ZnO. These heterostructures exhibit good stability because of the good compatibility between TiO_2_ and ZnO and similar band alignments [2]. Also, TiO_2_/ZnO heterostructures are expected to act as better photoanodes on account of the combination of the very high reactivity of TiO_2_ and large binding energy of ZnO [10, 11].

Several attempts are being made to fabricate heterostructures based on nanostructures of TiO_2_ and ZnO such as TiO_2_ nanotube/ZnO nanorod composite, ZnO-coated TiO_2_ nanotubes, and branched ZnO nanorod/TiO_2_ nanotube arrays due to the high surface-to-volume ratio attainable [12–17]. It has been found that the presence of ZnO prevents the fast recombination of the photogenerated charge carriers with the dye molecules in dye-sensitized solar cells because of the slightly higher band gap of the ZnO compared to the TiO_2_ [18, 19]. The extension of the photoresponse range and enhanced mobility of the charge carrier can also be expected from these heterostructures.

In the present work, heterostructure of ZnO nanoflakes partially covering the Zn-doped TiO_2_ nanotube (Zn-TONT) was fabricated for the first time. The layer of ZnO nanoflakes is expected to increase the dye absorption and the Zn doping of TiO_2_ nanotubes tends to increase their electrical transport properties. The improved performance of a dye-sensitized solar cell (DSSC) with Zn-TONT/ZnO nanoflake heterostructure instead of pure TONT as photoanode is also demonstrated.

## Experimental Methods

**Fig. 1:**
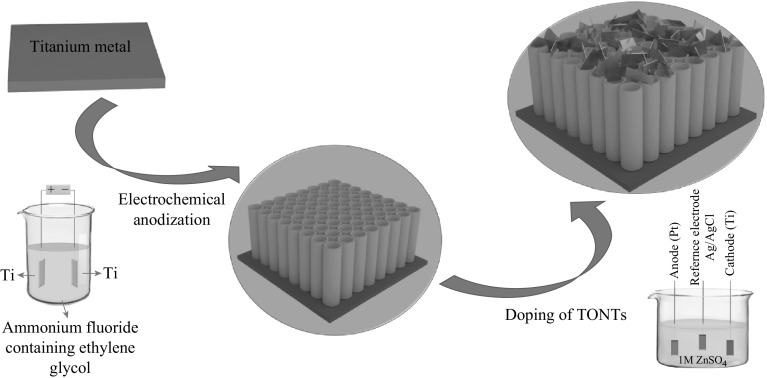
Schematic of the fabrication of Zn-TONT/ZnO nanoflake heterostructure

The Zn-TONT/ZnO nanoflake heterostructure was fabricated by a two-step method (Fig. 1). In the first step, well-aligned and uniform TONT were fabricated on titanium foil by electrochemical anodization [20]. In the second step, Zn doping and tailoring of ZnO nanoflakes on the Zn-TONT were done using a three-electrode system, where TONT was used as the working electrode, platinum rod as counter electrode, and Ag/AgCl as reference electrode (3 M KCl electrolyte), with 0.1 M ZnSO_4_ in ionized water as the solution for doping. A negative voltage pulse of 1–2.5 V was applied to the working electrode for duration of 2–10 s to trigger the doping process.

The detailed analysis of the Zn-TONT/ZnO nanoflake heterostructure was performed using X-ray diffractometer (XRD), field emission scanning electron microscopy (FESEM), Rutherford backscattering spectroscopy (RBS), and X-ray photoelectron spectroscopy (XPS). The current density–voltage (*J*–*V*) data of the DSSC were carried on a Solar Simulator (NEWPORT) under the standard solar conditions.

## Results and Discussion

**Fig. 2:**
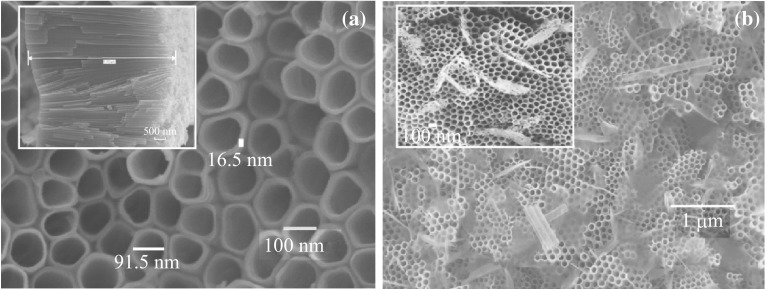
FESEM images of **a** *top view* of undoped TONT, and side view of undoped TONT (*inset*). **b** *Top view* of Zn-TONT/ZnO nanoflake heterostructure, and *top view* of the heterostructure after annealing at 500 °C (*inset*)

The FESEM surface images of the pure TONT and Zn-TONT/ZnO nanoflakes are shown in Fig. 2. Figure 2a indicates that the pure TONT formed by electrochemical anodization possesses an approximate inner diameter of 100 nm, wall thickness of 16 nm, and thickness of 6.9 µm. The side view of the as-formed aligned nanotubes is shown in the inset of Fig. 2a. Sponge-like ZnO nanoflakes (Fig. 2b) appear on the surface of TONT when doping was done for a short time of ~2 s, and partially cover the top of the tubes upon annealing, as seen in the inset of Fig. 2b.

**Fig. 3:**
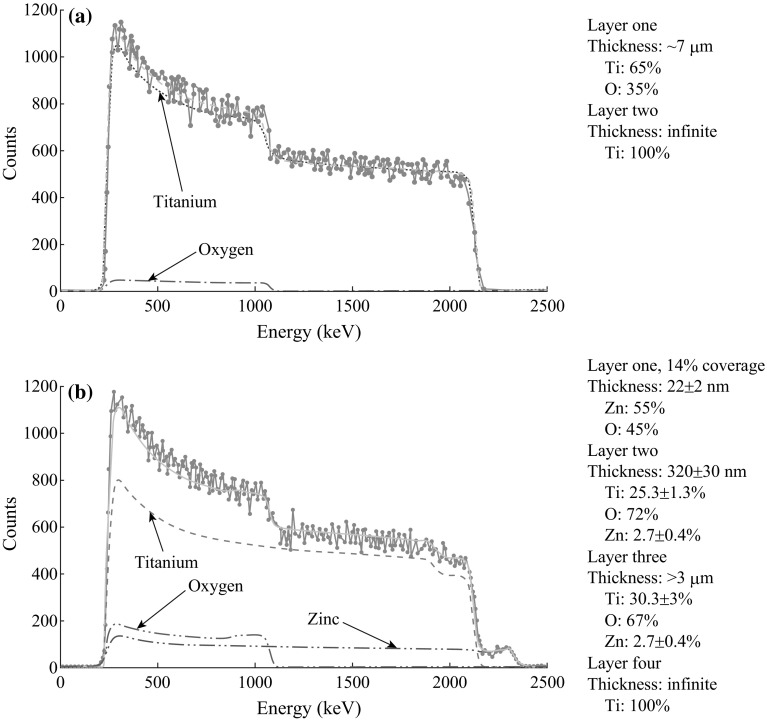
RBS of **a** pure TONT (areal thickness of 1.1 × 10^19^ atoms cm^−2^), and **b** Zn-TONT/ZnO nanoflake heterostructure (*Layer 1* areal thickness of 1.5 × 10^17^ atoms cm^−2^, *Layer 2* areal thickness of 2.9 × 10^18^ atoms cm^−2^, and *Layer 3* areal thickness of >3 × 10^19^ atoms cm^−2^)

More detailed layered structures of both pure TONT and the heterostructure were studied using RBS of 2.97 MeV α particles, a technique that can effectively bring out the at.% composition and thickness of different layers through SIMNRA fitting [21]. The RBS spectrum of pure TONT fitted with SIMNRA shows a structure of ~7 µm TiO_2_ as the top layer and a Ti metal substrate as the bottom layer (Fig. 3a). The spectrum from the Zn-TONT/ZnO structure suggests four layers (Fig. 3b), with a partial coverage by ZnO flakes at the top surface and a slightly Ti-depleted layer at the interface of the heterostructure. Partial coverage of ZnO flakes is simulated by superposing weighted models of ZnO-covered Zn-TONT and ZnO-uncovered Zn-TONT. The thickness of the ZnO flakes assuming ~14 % coverage of the top, is around 22 nm and the Zn-TONT beneath the ZnO layer consists of >3 µm with a Zn doping at.% of 2.7 ± 0.4. Between the TONT layer and the ZnO layer, the Ti-depleted layer (Ti: 25.3 ± 1.3 %) has a thickness of 320 ± 30 nm. The fourth and last layer is the Ti metal substrate with infinite thickness.

**Fig. 4:**
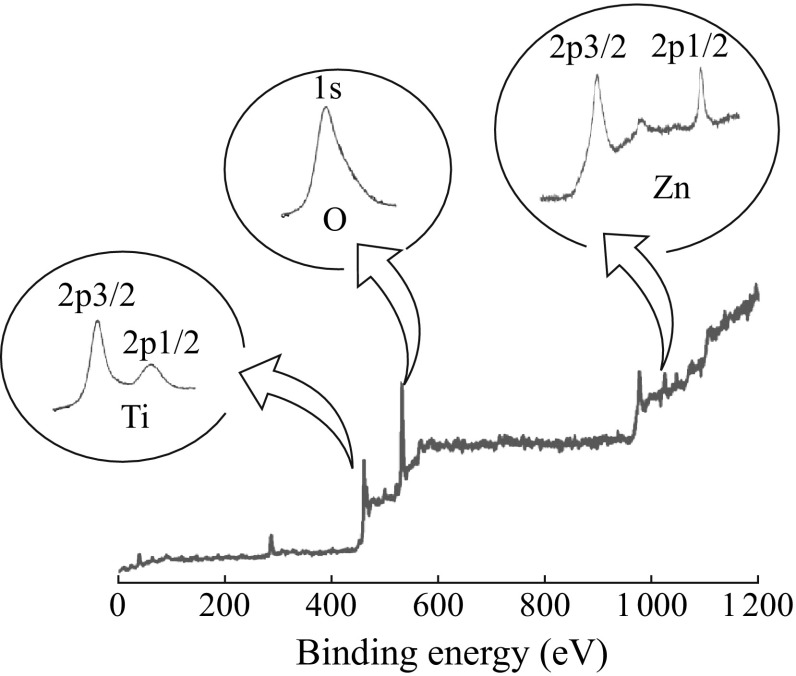
XPS of the Zn-TONT with 3 % zinc doping

RBS confirms that during the doping step, in addition to the formation of ZnO nanoflakes on top, a small percentage of Zn was doped into the TONT, the adsorption has led to a preferential orientation of the nanocrystallites in the tube on annealing. This phenomenon has been discussed in detail elsewhere [22]. The 3 % Zn doping of the TiO_2_ nanotubes was confirmed by taking the XPS of the heterostructure after removing the top ZnO nanoflake layer by dipping in 1 M HCl for one hour. The inset of Fig. 4 shows the detailed spectrum of the titanium, oxygen, and zinc, respectively.

**Fig. 5:**
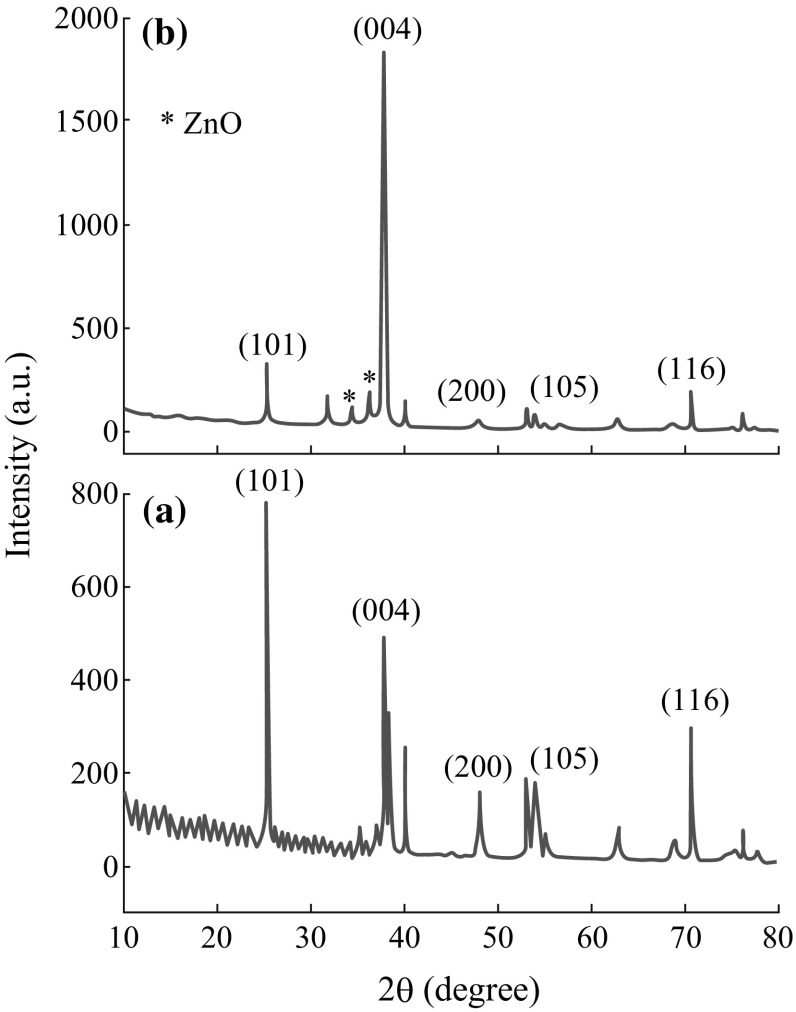
XRD patterns of **a** pure TONT, and **b** Zn-TONT/ZnO nanoflake heterostructure

The crystallinity of the annealed pure TONT and Zn-TONT/ZnO nanoflake heterostructure characterized by XRD (Fig. 5) reveals that the pure TONT (Fig. 5a) consist of (101), (004), (200), and (105) planes with preferential orientation along (101) plane (JCPDS 89-4203). Contrary to this observation, the TONT in the heterostructure (Fig. 5b) shows preferential orientation along (004) plane. This strong preferential orientation has been interpreted on the basis of the Zn-assisted minimization of the surface energy of (004) plane [22].

When Zn is doped into the tubes and then annealed, the amorphous TONT break up into crystallites and Zn gets preferentially adsorbed on the higher surface energy (0.90 J m^−2^) facets {001} which possess more adsorption ability. The Zn adsorption reduces the surface energy of the facet, resulting in the enhancement of the surface area, until the differential adsorption ability of that facet gets weaker and further adsorption and growth are impeded. There will be no further reduction in the surface energy of the {001} facets, and hence it does not go below the surface energy (0.44 J m^−2^) of {101} facets. During the stacking of the crystallites, the comparatively higher energy {001} facets connect each other and hence the {001} surfaces faces the tube up. The XRD peaks observed at 34.4° and 36.6° confirm the formation of the ZnO layer on the top of TONT (JCPDS-79-0205).

**Fig. 6:**
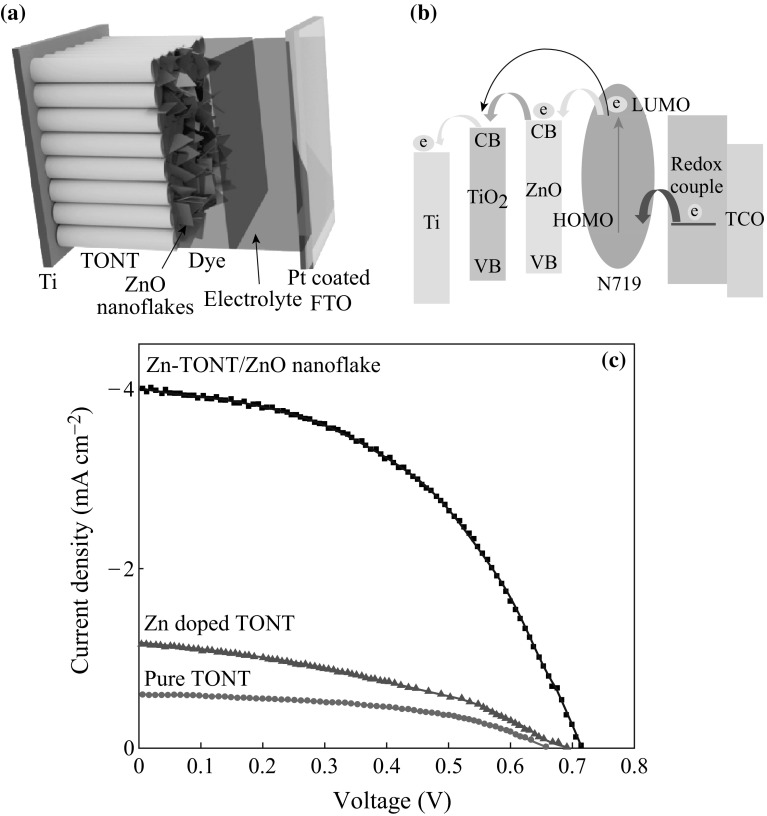
**a** Schematic of the DSSC with Zn-TONT/ZnO nanoflake heterostructure, **b** band structure of the DSSC fabricated using Zn-TONT/ZnO nanoflake heterostructure, and **c** *J*–*V* curves of DSSCs

The performance comparison of the TONT and the heterostructure Zn-TONT/ZnO nanoflake in DSSC application was done by fabricating both type of cells, one with TONT and the other with Zn-TONT/ZnO nanoflake heterostructure as the photoanode, and platinum-coated FTO as the front electrode. After dye sensitization of the photoanode with N719, a redox electrolyte was sandwiched between the photoanode and the front electrode for the regeneration of the dye molecules (Fig. 6a).

The *J*–*V* data of the DSSC are depicted in Fig. 6c. The *J*–*V* characteristics of the DSSC (Fig. 6c) using Zn-TONT/ZnO nanoflake heterostructure and the Zn-doped TONT after removing the top ZnO nanoflake layer are compared with that of DSSC using the as-prepared TONT. While the improvement in the open circuit voltage (*V*_oc_) with the heterostructure DSSC is only about 70 mV than that with pure TONT, the short circuit current for the heterostructure (*I*_Sc_ ~4 mA) is about an order of magnitude higher on comparison with the pure TONT (*I*_sc_ ~0.6 mA). The *V*_oc_ and *I*_sc_ of DSSC with Zn-doped TONT show a slight increase ~30 mV and 0.6 mA, respectively. This has produced a considerable increase (>7 times) in the efficiency of the heterostructure cell over the pure TONT-based cell, fabricated and operated under the same conditions, while the efficiency of DSSC with Zn-doped TONT over that of pure TONT is ~2 times. The result shows that although efficiency is slightly increased in the DSSC using Zn-TONT in comparison with that using pure TONT, the DSSC fabricated with Zn-TONT/ZnO nanoflake heterostructure shows still a significant enhancement when compared to either of them. Hence, it can be concluded that the improved performance is due to the combined effect of ZnO nanoflake and preferential [001] orientation of the anatase TONT, where the effect of ZnO nanoflakes is more prominent than the orientation effect. The results emphasize the significance of TONT/ZnO photoanodes over pure TONT photoanodes, for improved DSSC performance.

The improvement in the efficiency of DSSC could be explained based on the more effective processes of carrier production and transport in the heterostructure-based DSSC whose band alignment is illustrated in Fig. 6b. In TONT-based DSSC, when the solar radiation falls on the dye molecules through the transparent platinum FTO window and the electrons from the HOMO (highest occupied molecular orbital) of the N719 dye are excited to the LUMO (lowest unoccupied molecular orbital), the photogenerated electrons are injected into the conduction band of the TONT and are collected by the Ti metal back contact. In this process, there is a large possibility for the charge recombination of the electrons in the conduction band of the TONT and holes present in the HOMO of the dye, which may reduce the efficiency of the device. In the Zn-TONT/ZnO nanoflake heterostructure-based DSSC, the presence of ZnO nanoflakes (band gap ~3.37 eV with conduction band and valence band positioned slightly above that of the corresponding bands in TONT) on the top of the TiO_2_ nanotubes (band gap ~3.2 eV) decreases the recombination rate of the electrons owing to the small energy barrier created by them [19, 23]. In addition, ZnO lattice was reported to provide electron mobility almost 3 times larger compared to the TONT which facilitates faster transport of the generated electrons to the back metal contact [18]. This increased mobility thus acts as an additional factor that further reduces the recombination rate of the photogenerated charge carriers. This synergetic property of Zn-TONT/ZnO nanoflakes in promoting efficient separation of carriers may be the reason for improved solar cell efficiency.

## Conclusion

In conclusion, a clear picture of the layered structure, morphology, and crystallinity of the Zn-TONT/ZnO heterostructure was gained using RBS, SEM, and XRD. The advantage of using heterostructures of the Zn-TONT/ZnO nanoflake heterostructure in a DSSC constructed by a cost-effective and highly reproducible method was analyzed. This layered heterostructure shows enhanced DSSC efficiency over the pure TiO_2_ nanotubes.
